# Multi‐Degree‐of‐Freedom Robots Powered and Controlled by Microwaves

**DOI:** 10.1002/advs.202203305

**Published:** 2022-08-19

**Authors:** Yongze Li, Jianyu Wu, Peizhuo Yang, Lizhong Song, Jun Wang, Zhiguang Xing, Jianwen Zhao

**Affiliations:** ^1^ Department of Mechanical Engineering Harbin Institute of Technology Weihai 264209 China; ^2^ School of Information Science and Engineering Harbin Institute of Technology Weihai 264209 China

**Keywords:** microwave directional heating, microwave‐driven robot, passive robot, SMA actuator

## Abstract

Microwaves have become a promising wireless driving strategy due to the advantages of transmissivity through obstacles, fast energy targeting, and selective heating. Although there are some studies on microwave powered artificial muscles based on different structures, the lack of studies on microwave control has limited the development of microwave‐driven (MWD) robots. Here, a far‐field MWD parallel robot controlled by adjusting energy distribution via changing the polarization direction of microwaves at 2.47 GHz is first reported. The parallel robot is based on three double‐layer bending actuators composed of wave‐absorbing sheets and bimetallic sheets, and it can implement circular and triangular path at a distance of 0.4 m under 700 W transmitting power. The thermal response rate of the actuator under microwaves is studied, and it is found that the electric‐field components can provide a faster thermal response at the optimal length of actuator than magnetic‐field components. The work of the parallel robot is demonstrated in an enclosed space composed of microwave‐transparent materials. This developed method demonstrates the multi‐degree‐of‐freedom controllability for robots using microwaves and offers potential solutions for some engineering cases, such as pipeline/reactors inspection and medical applications.

## Graphical abstract

In addition to providing energy, wireless‐driving systems using microwaves can potentially offer unique control information based on the polarization direction and frequency. Here, a far‐field microwave‐driven parallel robot controlled by adjusting energy distribution via changing the polarization direction of microwaves at 2.47 GHz is first reported. The robot can realize circular path and triangular path motions.

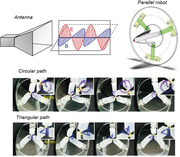

## 1 Introduction

Wireless‐driving robots have been widely studied because the needless of the batteries, cables, electronic circuits, etc. These characteristics meet the requirements about lightweight, miniaturization, and controllability of microrobots.^[^ ^1^, ^2^, ^3^, ^4^ ^]^ There have been some physical fields used in wireless power transfer (WPT), including magnetic, optical, ultrasonic, and electric (**Table** **1**), microwaves among them have gradually become a popular WPT strategy with numerous application prospects in military,^[^ ^5^ ^]^ industrial,^[^ ^6^ ^]^ civilian^[^ ^7^ ^]^ fields, etc., because of their excellent capabilities of far‐field transmission, penetrating obstacles, wave‐targeting/focusing, information carrying, and nonmechanical steering via phased array antennas. In addition to providing energy, wireless‐driving systems using microwaves could potentially offer unique control information by the polarization direction and frequency of the microwaves compared to other static‐field‐based wireless actuation methods. Moreover, microwaves could flexibly and quickly realize polarization direction control through phased array technology.

**Table 1:** Brief evaluation of various untethered driving methods for robots

Category	Actuation method	Advantages for applications	Application scenarios	Major limitations
Magnetic	Quasi‐static magnetic fields	High response rate; high accuracy; better penetration; security of human–computer interaction^[^ ^19^, ^20^ ^]^	Targeted drug delivery;^[^ ^21^, ^22^, ^23^ ^]^ cell culture;^[^ ^24^, ^25^ ^]^ microassemblies;^[^ ^26^, ^27^ ^]^ noninvasive medical interventions in the vascular system^[^ ^28^, ^29^ ^]^	Difficulty in selective agent addressability;^[^ ^26^ ^]^ complex and expensive external equipment^[^ ^30^ ^]^
Electric	Electric field gradient	Simple drive equipment; low power consumption^[^ ^31^ ^]^	Drug screening; disease treatment;^[^ ^32^ ^]^ artificial muscle^[^ ^33^ ^]^	Slow response; limited to solution environments^[^ ^34^, ^35^ ^]^
Acoustic	Microbubbles^[^ ^36^ ^]^ or pressure gradients^[^ ^37^ ^]^	Good compatibility and gentleness with biological samples and environmental fluids;^[^ ^38^ ^]^ both 2D and 3D assemblies can be realized^[^ ^39^ ^]^	Disease diagnosis;^[^ ^40^ ^]^ genetic analysis; drug screening and therapy^[^ ^41^ ^]^	Limited drive power; no suitable driving device has been obtained in vivo^[^ ^42^ ^]^
Humidity	Exploitation of swelling due to humidity gradients^[^ ^43^, ^44^ ^]^	Propel itself without any artificial energy	Military and environmental robots; bio‐robot;^[^ ^45^, ^46^ ^]^ biomedical treatments	Close driving distance; less freedom
Light	Light‐induced formation^[^ ^47^, ^48^, ^49^, ^50^ ^]^	Long drive distance;^[^ ^51^ ^]^ good directionality^[^ ^52^ ^]^	Environmentally responsive soft robotic systems;^[^ ^53^, ^54^ ^]^ cargo delivery^[^ ^55^, ^56^ ^]^	Not applicable in opaque conditions
Microwave	Thermally drive	Selective heating; Integral uniform heating	Microwave exposure environment,^[^ ^10^ ^]^ medical care^[^ ^9^, ^57^ ^]^	Damage to human health and electronic circuits

Due to the potential advantages mentioned above, microwave powered artificial muscles have gradually attracted attention to work for healthcare^[^ ^8^, ^9^ ^]^ or to replace humans working in high‐level microwave exposure environments^[^ ^10^ ^]^ (radio and television transmitting stations, radar stations, etc.), which have great damage on human health, electrocommunication, and circuit systems. Microwaves usually exhibit three characteristics to substances: penetration, reflection, and absorption. Several materials, such as plastics, ceramics, paper, and some polymers, are transparent to microwave energy effectively. Metals have high reflection properties, while substances with large dielectric loss factors, such as alcohol and water, have a strong ability to absorb microwaves. Microwave powered artificial muscles mainly utilize substances that can generate Joule heat or dielectric loss in materials, such as carbon nanomaterials,^[^ ^8^, ^11^, ^12^ ^]^ iron oxide powder,^[^ ^13^ ^]^ alcohol,^[^ ^9^ ^]^ etc., to induce the shape memory effect,^[^ ^14^ ^]^ gas–liquid phase transition, and anisotropic thermal expansion^[^ ^10^ ^]^ to achieve muscle deformation. Compared with other thermal driving methods through hydrothermal,^[^ ^15^ ^]^ hot air,^[^ ^16^ ^]^ photothermal,^[^ ^17^ ^]^ or current,^[^ ^18^ ^]^ microwaves offer several advantages, like selective heating, far heating, and uniform heating in material volume.

Although there have been some studies on the actuation mechanisms and structures of microwave actuators, the control for the multi‐degree‐of‐freedom (MDOF) robot by a single adjustable microwave source has not yet been achieved because of the following two challenges: first, the response of the actuator should be yare enough for practical use in the far‐field, but not just in the microwave oven; second, the overall actuation system is hard to control by a single transmitting system utilizing the characteristics of microwaves, which would simplify the complexity of the mechanical system while increasing the discrimination of microwaves in ambient electromagnetic interference. Microwave, indeed not only is energy source, but also is carrier in the propagation, such as frequency, phase, and polarization direction. Thus, it is potential to realize MDOF control of robotic systems by decoding the carrier information in microwaves.

**Figure 1:**
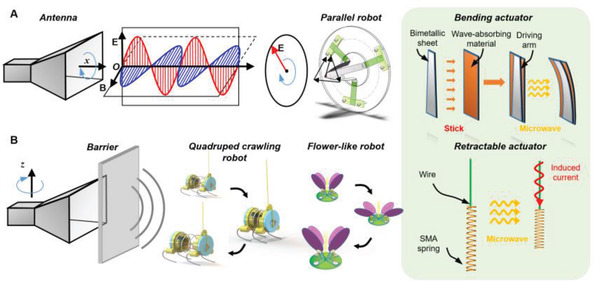
Microwave‐driven robots with two different motion forms. A) Schematic diagram of the microwave‐driven‐parallel robot with bending actuators powered and controlled by ambient microwaves. By rotating the horn antenna around the *x*‐axis to control the polarization direction of the ambient microwave, the received microwave power of each bending actuator can be adjusted. B) Schematic diagram of the quadruped robot and flower‐like robot with retractable actuators activated by ambient microwave. The on–off control of the robots can be achieved by rotating the horn around the *z*‐axis or adjusting the polarization direction of the microwaves.

In this article, we report a microwave‐driven parallel robot that can realize circular and triangular path control by adjusting the direction of microwave polarization at 2.47 GHz in the far‐field. The far‐field range from 291 mm to infinity can be obtained by the geometry of the wave port and wavelength of the microwave (Text S1, Supporting Information). Three double‐layer lamelliform microwave‐driven bending actuators, which are made by wave‐absorbing material and bimetallic strip (5J20110) work as active arms of the parallel robot (**Figure** **1**A). The wave‐absorbing material is a high‐bandwidth commercial soft electromagnetic absorbing film filled with ferromagnetic alloys (CAS no: 7439‐89‐6, Shanghai Xinxi Alloy Materials Co., Ltd.) that can absorb the external electromagnetic energy and act as volumetric heating sources for the bimetal. In order to reduce the reflection of microwaves by the bimetal strip, the shape of the bimetal is a trapezoid whose area is 50% of the area of the wave‐absorbing material, and the driving power of the microwave will not cause the bimetal to spark. Through the combination of two commercial materials used in different fields, the microwave‐driven actuator is realized, which has a low cost due to the accessible materials and simple fabrication process.

To have a sufficient response rate for the bending actuator, we studied the optimal length of the wave‐absorbing sheet, and it is found that it can rise to 80 °C within 10 s under the radiation power of 700 W outside 0.4 m. This is sufficient for occasions where the speed requirement is not very high. On the parallel robot, three bending actuators with 120° intervals have different receiving efficiencies for microwaves with different polarization directions. Through changing the polarization direction of the microwave, the response of each bending actuator can be adjusted, and the circular and triangular paths of the end point of the parallel robot can be realized. A theoretical model was also developed to guide the path control of the parallel robot. In addition, we also verified the working effect of the parallel robot in a confined space covered by microwave‐transparent but light‐opaque walls.

Also, in order to demonstrate the potential of microwave‐driven robots in miniaturization, lightweight, flexible motion, and swarm‐driving, a quadruped crawling robot and a flower‐like robot based on retractable actuators are designed (Figure 1B), where the wire acts as a microwave receiver and the shape memory alloy (SMA) spring acts as an actuator. SMAs are capable of sustaining large inelastic strains that can be recovered at phase‐transition temperatures. Compared with the SMA wire (the shrinkage rate is about 3–5%), the SMA spring has greater linear movement ability (the shrinkage rate is about 40%).^[^ ^58^ ^]^ Moreover, SMA actuators have the highest power density and stress^[^ ^59^ ^]^ compared to other artificial muscles such as piezoelectric actuators, dielectric elastomer actuators, soft fluid actuators, shape memory polymer actuators, and ionic polymer‐metal composite actuators. The quadruped crawling robot is about 15 mm in length, weighs only 0.42 g, and has a driving distance of 0.4 m, which is currently the lightest radiofrequency(RF)‐powered robot.^[^ ^60^ ^]^ The flower‐like robot, which has four actuators, can mimic the nastic movement under the radiation of microwaves. The far‐field microwave‐driven (MWD) parallel robot can be used in an enclosed container where the other driving components are not workable, such as controlling a reaction that requires ultrahigh pressure. In addition, other types of MWD robot can be used in medical treatments, such as performing integrated radiofrequency ablation in vivo.

## 2 Results

### 2.1 Microwave Energy to Thermal Energy of Wave‐Absorbing Sheet: Principle and Characteristic

**Figure 2:**
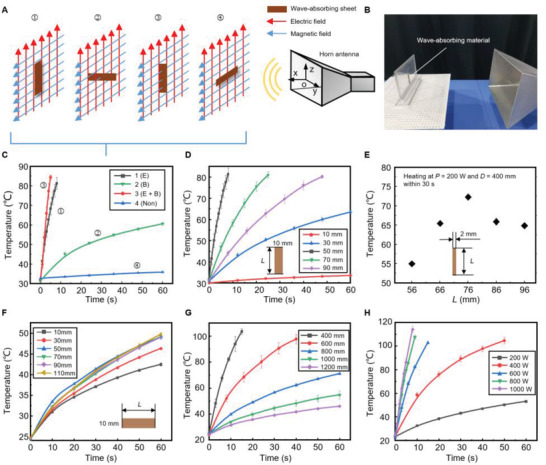
Wave‐absorbing sheets under the microwave. A) Schematic showing poses of the sheet in the microwave field with the serial numbers labeled on the top. B) Layout of the testing platform. C) The experimental results show the heating effect of the wave‐absorbing sheet with different poses in the microwave field. D) Temperature increases in the sheet with different *L* values (insets) at pose 1, where *P* = 700 W and *D* = 400 mm. E) Temperature versus the length *L* of a 2 mm width slender microwave‐absorbing material after heating 30 s in microwaves (*P* = 200 W and *D* = 400 mm). Error bars indicate the SD for *n*   =   3, where *n* is the number of groups of experimental data. F) Temperature rise in the sheet with different *L* values (insets) at pose 2, where *P* = 700 W and *D* = 400 mm. G,H) The temperature rise in the sheet at pose 1 under different transmitting powers *P* and different distances *D* between the wave‐absorbing sheet (50 mm × 10 mm) and the wave port. Error bars in (C, D, and F to H) indicate the SD for *n*  =   6.

The bending actuator in the parallel robot is a double‐layer structure driven by the thermal effect of microwaves in which the wave‐absorbing material layer converts the microwave energy into volume heat. Therefore, the characteristics of wave‐absorbing materials in microwave fields are crucial for the optimization of bending actuators. According to the analysis, the heating effect of the wave‐absorbing material in the microwave is related to its posture, size, distance, and microwave power. According to the received microwave components, the poses of the wave‐absorbing sheet in the microwave field can be divided into four types, which are labeled with serial numbers on the top of **Figure** **2**A. In the far‐field of the microwave radiated from the pyramidal horn antenna, the direction of the electric field and magnetic field are parallel to the width (*z*‐axis) and length (*y*‐axis) of the wave port, respectively (Figure 2A). The wave‐absorbing sheet in different poses within the space field will produce heat loss through different mechanisms (e.g., joule heat, magnetic hysteresis loss, dielectric loss, and eddy). In pose 1, the length direction of the sheet is parallel with the electric field while the width direction is parallel with the magnetic field; therefore, the heating effect mainly depends on the electric field. In pose 2, the width direction of the sheet is along the electric field and its thickness direction is along the magnetic field; therefore, the heating effect mainly depends on the magnetic field. Using the same analysis, in pose 3, both the electric field and magnetic field contribute to heating the sheet; in pose 4, both the electric field and magnetic field are almost useless for heating. The photo of the device is shown in Figure 2B (see Figure S1 in the Supporting Information for details).

To study the heating characteristics of wave‐absorbing sheets in different poses within the microwave field, we recorded the temperature of a 50 mm × 10 mm wave‐absorbing sheet in the microwave field with an infrared thermal imager (FLUKE TiS50, Fluke Corporation), as shown in Figure 2C. The distance *D* between the wave‐absorbing sheet and the wave port was 400 mm, which was the far‐field of the antenna (Text S1, Supporting Information), and the transmitting power *P* was 700 W. Figure 2C shows the heating effect on a 50 mm × 10 mm wave‐absorbing sheet under the electric field, magnetic field, or both. Under the action of the electric field, the wave‐absorbing material can rise to 80 °C in 10 s, while under the action of the magnetic field, it can reach about 60 °C in 1 min, which means that the electric field contributed a greater amount of thermal energy than the magnetic field did. Moreover, comparing curve 3 and curve 1, the temperature rise rate reached the highest when both electric and magnetic fields impact on the sheet, indicating a positive superposition of the electric and magnetic fields for the thermal effects on the wave‐absorbing sheet. In curve 4, the temperature of the sheet was almost unchanged, indicating that both fields barely contributed to heating.

The wave‐absorbing sheet used in this study is an ultra‐thin magnetic alloy composite material (Table S1, Supporting Information), whose dimensional characteristics affect the microwave absorption efficiency. As shown in Figure 2D, the thermal efficiency of the wave‐absorbing sheet at pose 1 changed dramatically with the length *L* and reached a maximum value at 50 mm. According to the Fries transfer formula, the received power *P*_r_ of the wave‐absorbing sheet is

(1) $$P_{r}=P_{t}\frac{G_{t}G_{r}\lambda^{2}}{{\left(4\pi D\right)}^{2}}$$

where *P*_t_ is the transmitted power of the microwave, *G*_r_ is the gain of the receiver, and *G*_r_ = *η*_rad_ *D*_m_, *η*_rad_ is the radiation efficiency of the receiving antenna, *D*_m_ is the directivity of the receiving antenna, *G*_t_ is the gain of the transmitting antenna, *λ* is the wave‐length of the microwave, and *D* is the transmission distance. When the wave‐absorbing sheet only receives the electric field, it can be approximated as a monopole antenna, whose gain *G*_r_ is related to its length. According to the dipole antenna theory, when the arm length, which is equal to the length *L* of the monopole antenna, is 0.625 *λ* (76.25 mm), the directivity is the largest. For a monopole antenna on infinite ground, the directivity coefficient is proportional to and twice that of a dipole antenna, so it also reaches a maximum value at 0.625 *λ*. However, due to the geometry difference between the sheet‐like wave‐absorbing material and the line‐like antenna, the length of the maximum gain is shifted. To verify the effect of the geometry of the wave‐absorbing sheet on the optimal length, we measured the temperature versus the length *L* after microwave heating for 30 s for an elongated wave‐absorbing strip (width = 2 mm, *P* = 200 W, and *D* = 400 mm). The geometrical characteristic of the strip is more similar as a monopole antenna (Figure 2E). Experimental results show that the length of the maximum received power of the wave‐absorbing strip is equal to the theory value of the monopole, indicating that the thermal mechanism of the wave‐absorbing strip under the electric fields is the Joule heat generated by the alternating current. However, the optimal length of the wave‐absorbing sheet with a width of 10 mm decreased to 0.42 *λ* due to the equivalent inductance on it.

When the wave‐absorbing sheet is only heated by the magnetic field (pose 2, *P* = 700 W, *D* = 400 mm), the heating mechanisms are mainly hysteresis loss and eddy current loss. According to Figure 2F, when the length *L* (inset) is less than 50 mm, the heating efficiency of the wave‐absorbing sheet is proportional to *L*; whereas, the temperature increase rate is basically unchanged when *L* is over the half‐wavelength (60 mm). The result shows that the maximum size of a single eddy current is equal to half‐wavelength, so when *L* is less than half‐wavelength, the eddy current will decrease (Figure S2, Supporting Information).

Figure 2D,F shows that, when the electric and magnetic fields worked on the wave‐absorbing sheet simultaneously (pose 3), *L* determined which component of the microwave plays a key role in the thermal effect. For example, when *L* is 10 mm, the magnetic field is the key contributor to the thermal effect; when *L* is 50 mm, the temperature rise is primarily due to the electric field; when *L* is 30 mm, the magnetic field and the electric field have the same thermal effect on the sheet.

The Poynting vector of microwaves radiated from the antenna decreased gradually with the increase of transmission distance *D* (Equation (1)), thus the temperature rise rate of the wave‐absorbing sheet (pose 1, *P* = 700 W) would also decrease with the increase of *D* (Figure 2G). Figure 2G shows that when the distance between the wave‐absorbing material and the wave port exceeds 800 mm, the time of the temperature rise of the wave‐absorbing material to 60 °C is about 40 s. The slow temperature response rate of wave‐absorbing materials will limit the practical application and motion range of microwave actuators. Therefore, considering the practical application of microwave actuators, the maximum control distance for the microwave‐driven robot is 800 mm.

Figure 2H shows the relationship between the temperature rise rate of the wave‐absorbing sheet and the microwave power *P* (*D* = 400 mm, pose 1). The temperature rise rate gradually increases as *P* increases, and when the power is above 600 W, the temperature increases to 100 °C from the room temperature within 10 s. The dimension of the sheets in Figure 2G,H is 50 × 10 mm. For the reason of the instrument itself, we discarded the calibration temperature point of the instrument. In conclusion, we studied the important contribution of the electric field in microwave‐driving and optimized the length of the wave‐absorbing material under the action of the electric field. Based on the above studies, we designed a bending actuator and applied it to an MWD parallel robot.

### 2.2 Controllability of the Robot Driven by Microwaves

**Figure 3:**
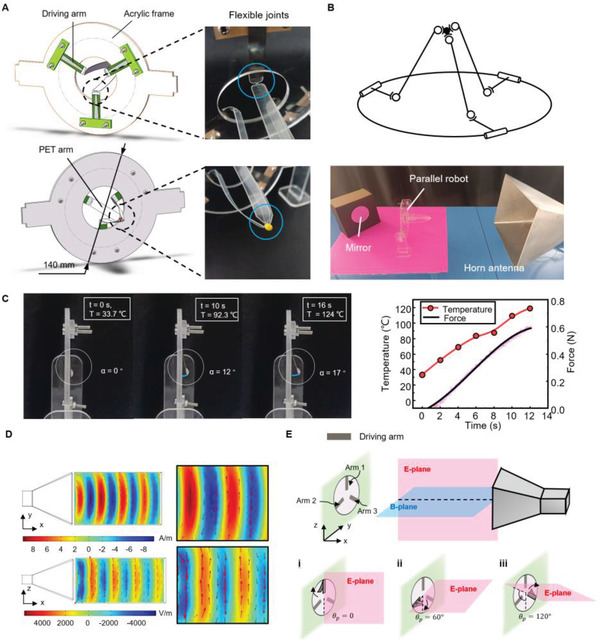
Parallel robot controlled by microwaves. A) Schematic showing a parallel robot composed of three sections: three driving arms, an acrylic frame, and a PET arm. The PET arm has six flexible joints (right). B) Kinematical diagram of the parallel robot (top) and the location of the robot relative to the horn antenna (bottom). C) Sequence of images showing the characteristics of the driving arm under microwave irradiation (left) and the relationship between temperature and force (right). D) Simulation results of the magnetic (top) and electric (bottom) field radiated from a horn antenna using COMSOL. E) Schematic diagram of controlling the parallel robot via tuning the direction of polarization.

Based on the principle of microwave‐driving, all the parameters that affect the conversion of microwave energy into thermal energy can be used to control the received power of MWD components, such as the polarization direction, frequency, and power of microwave. To verify this deduction, we designed an MWD parallel robot controlled by the polarization direction of microwaves. As shown in **Figure** **3**A, the whole parallel robot was consisted of three sectors: 1) three bending actuators consisted of the driving arms. 2) A polyethylene terephthalate (PET) arm with six flexible joints consisted of driven arms and moving platform. 3) An acrylic sheet consisted of the fixed platform. As shown in the figure, one side of three driving arms was fixed on the acrylic frame at 120° intervals, and the other side was bonded to the PET arm (see the Experimental Section for details). The parameters of the parallel robot: the diameter of the fixed platform = 100 mm; the diameter of the moving platform = 3 mm; the length of the driving arm = 40 mm; the length of the slave arm = 90 mm. The projected length *l_pi_* (*i* = 1, 2, 3) of each bending actuator in the polarization direction is

(2) $$l_{pi}=l_{a}\cos\theta_{i},i=1,2,3,\left(\theta_{1}+\theta_{2}+\theta_{3}=2\pi \right)$$

where *l*_a_ is the length of the bending actuator, *θ*_*i*_ is the angle between the length of the bending actuator and the polarization direction. Based on experiment and analysis in Section 2.1, it can be derived that the heating rate will reach the maximum when the length of the bending actuator *L* is 50 mm. Corresponding to the principle of reducing the size as much as possible while ensuring the microwave receiving efficiency, here we set *L* to 40 mm.

As shown in Figure 3B (top), the equivalent kinematical diagram of the parallel robot could be seen as a 3‐RSS parallel robot. The moving platform could be equivalent to a point due to the size of the moving platform was much smaller than that of the fixed platform; thus, the robot had three translational DOF. Based on the experimental results, we can use both electric and magnetic fields in the microwave to produce thermal energy. Furthermore, to improve the controllability of the parallel robot, we placed the parallel robot in the position shown in Figure 3B (bottom). Thus, the increase in the temperature of the driving arm only depended on the electric field, and the received power of the wave‐absorbing sheet was related to the polarization direction of the microwave.

We measured the characteristics of the bending actuator (driving arm) at pose 1 with *P* = 700 W and *D* = 400 mm (Figure 3C). The bending angle of the actuator could reach 17° within 16 s, at the same time, the wave‐absorbing material had reached its maximum temperature for normal operation (120 °C). We also measured the relationship between the temperature and the pull force generated on the tip of the bending actuator. The result in Figure 3C (right) showed that the tip of the bending actuator weighed 0.68 g and generated a force of about 0.6 N at the ultimate temperature. The tension measuring device of the bending actuator is available in Figure S3 in the Supporting Information.

To realize the control of the polarization direction of microwaves in space, we designed a horn antenna that could rotate along the normal direction of the wave port. The result of simulation using COMSOL showed that the Poynting vector was along the normal direction of the wave port (*x*‐axis), the magnetic field was along the length direction (*y*‐axis) of the wave port, and the electric field was along the width direction of the wave port (*z*‐axis) (Figure 3D). The red arrows represented the orientation of the field vector, and the magnitude of the arrows was proportional to the field strength (see Figure S4 in the Supporting Information for details). The E‐plane (pink plane) of the microwave was parallel to the wide side of the wave port, and the B‐plane (blue plane) was parallel to the long side of the wave port (Figure 3E). The range of the microwave field of the horn antenna is available in Text S1 in the Supporting Information. Here, we define *θ*_p_ as the angle between the E‐plain and the vertical line (Figure 3E, bottom, drawn by the dotted line), and define $\lambda_{p}=\frac{l_{p}}{l_{a}}\;$, where *l*_a_ is the length of the driving arm and *l*_p_ is the projected length of the driving arm on the E‐plane. When the *θ*_p_ is equal to 0°, the projected length of the Arm 1 is equal to the original length (*λ*_p1_ = 1) and the received power is the largest. Whereas the projected length of Arm 2 and 3 is half of the origin length (*λ*_p2_ = *λ*_p3_ = 0.5) and the received power is less than that of Arm 1. Thus, the temperature of Arm 1 would rise significantly compared with the temperatures of the other two driving arms, generating significant bending (see Figure 3E‐i). When the *θ*_p_ was equal to 60° and 120° (see Figure 3E‐ii,iii), the principle was the same as previous analysis. Therefore, we could change the bending angle of the driving arm via controlling the *λ*_p_ by rotating the E‐plane.

We experimentally demonstrated the circular and triangular path control of the parallel robot via tuning the polarization direction of microwaves. Since microwaves interfered with the electronic equipment, it was difficult to photograph directly the front side of the parallel robot. Therefore, we placed a mirror on the back of the robot at a 45° angle (Figure 3B, bottom), and captured the motion path of the endpoint from the right side of the robot.

**Figure 4:**
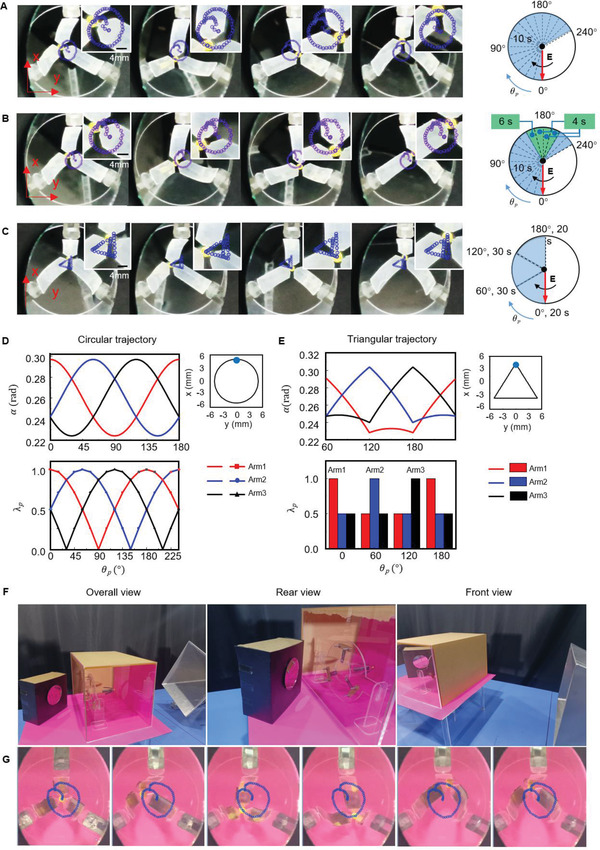
Path control for the microwave‐driven‐parallel robot. A) Photographic sequence of the parallel robot performed a circular path (left) under a unified change of the *θ*_p_ with step sizes equaled to 15° and 10 s intervals (right); refer to Movie S1 in the Supporting Information. The robot was 450 mm away from the antenna and the power of magnetron was 700 W. B) Photographic sequence of optimized circular path motion of the parallel robot (left) and the graph of the transform of *θ*_p_ during the motion (right). C) Photographic sequence of the robot performing a triangular path (left) under the variation of the *θ*_p_ (right); refer to Movie S2 in the Supporting Information. D) Inverse solution (top) and the variations in *λ*_p_ (bottom) when the parallel robot performed a circular path. The desired path is shown on the right side with a starting point (blue point) on the top. Here, *α* is the bending angle of the driving arm. E) Inverse solution (top) and the variation in *λ*_p_ (bottom) when the parallel robot performed a triangular path. The desired path of the inverse solution is shown on the right side. F) Photo of the parallel robot in a closed acrylic box. The robot was 450 mm away from the antenna. G) Image sequence showing the motion of the parallel robot in the confined space with the circular path.

**Figure** **4**A shows a photographic sequence of the circular path motion of the parallel robot (left) under the variation of *θ*_p_ (right) with *P* = 700 W and *D* = 450 mm (see Movie S1, Supporting Information). When the *θ*_p_ transformed from 0° to 240° in every 15° steps within 10 s intervals, the endpoint (yellow point) of the parallel robot moved approximately along a circular path (Figure 4A, left) from the center point. The path tracking error between the actual trajectory and the ideal trajectory is caused by the uneven power distribution. We observed that the radiation intensity of the antenna was stronger at 180° than in other angles. However, these effects could be weakened by adjusting the time interval for each step. As shown in Figure 4B (right), we shortened the time interval around *θ*_p_ = 180° (green sectors) and then the parallel robot produced a more ideal circular path (Figure 4B, left). In addition, Figure 4C (left) shows the photographic sequence of the triangular path motion of the parallel robot (see Movie S2, Supporting Information) under the following control logic: *θ*_p_ varied from 0° to 180° in every 60° step (Figure 4C, right). The time interval for each step in the control logic is determined experimentally.

To further investigate the control principle of the parallel robot based on bending actuators, we obtained its inverse solution (see Text S2, Supporting Information). Figure 4D (top) shows the inverse solution of the parallel robot with a desired path. The starting point was the blue point, and the *z*‐coordinate was equal to −80 mm, as shown on the right side. In the circular path shown in Figure 4A, when the *θ*_p_ varied from 0° to 240°, the *λ*_p_ of the three driving arms was shown in Figure 4D (bottom). Since the step size of *θ*_p_ was small, *λ*_p_ could be approximated as a continuous function. By comparison, it can be seen that *α* is proportional to *λ*_p_, which can be explained as follows.

The temperature change Δ*T* of the absorbing material in time Δ*t* is

(3) $$\begin{array}{ccc} \Delta T & = & \frac{1}{Cm}\left(Q_{h}-Q_{d}\right)\\ & = & \frac{1}{Cm}\left(\int P_{h}dt-\int P_{d}dt\right)\\ & = & \frac{\Delta t}{Cm}\left(\bar{P_{h}}-\bar{P_{d}}\right) \end{array}$$

where Δ*Q* is the quantity of heat received by the wave‐absorbing sheet, *C* and *m* are the specific heat capacity and mass of the wave‐absorbing sheet, respectively, and can be considered as constants. *P_h_* is the heating power of microwaves, *P_d_* is the dissipation power of heat on the driving arm, and ${\bar{P}}_{h}$ and ${\bar{P}}_{d}$ are the average value of *P_h_* and *P_d_* in time Δ*t*, respectively. Here, we only consider convective heat transfer losses; therefore, refer to Newton's law of cooling

(4) $$\bar{P_{d}}=hA\left(T-T_{0}\right)$$

where *h* is the convective heat transfer coefficient (W m^−2^ K^−1^), *A* is the superficial area of the driving arm (m^2^), *T* is the temperature of the driving arm (°C), and *T*_0_ is the environment temperature (°C).

As we all know, the maximum received power *P*_r_ (mW) of the receiving antenna is

(5) $$P_{r}=\frac{E_{0}^{2}c^{2}}{480\pi^{2}f^{2}}D_{r}$$

where *E*_0_ is the electric field strength (µV m^−1^), *f* is the frequency of electromagnetic waves (2465 MHz), *D*_r_ is the directivity coefficient of the receiving antenna, and *c* is the speed of light (3 × 10^8^ m s^−1^).

When the wave frequency *f* and the position of the wave‐absorbing sheet are fixed, *E*_0_ can be determined. Thus, the received power *P*_r_ is only related to *D*_r_. In this paper, the projected length of the wave‐absorbing sheet in the electric field varied from 0 to 40 mm. According to the characteristics of the monopole antenna, when the length of the wave‐absorbing sheet is shorter than half‐wavelength, *D*_r_ is approximately proportional to *λ*_p_; therefore, *P*_r_∝*D*_r_∝*λ*_p_. So,

(6) $$\bar{P_{h}}=P_{r}=k\lambda_{p}$$

where *k* is a coefficient to be determined.

The bimetal sheet on the parallel robot is a cantilever beam structure. When the temperature of the bimetal sheet rises to *T*, the rotation angle *α* of its end is

(7) $$\alpha =\frac{2K(T-T_{0})}{\delta }l$$

where *K* is the specific thermal deflection of the bimetal sheet (1 °C^−1^) and it is a constant in the linear temperature range (−20 to 150 °C), *l* is the length of the bimetal sheet that can bending (mm) and *δ* is the thickness of the bimetal sheet (mm). Substitute Equations (4), (6), and (7) into Equation (3) to get

(8) $$Cm\frac{\Delta T}{\Delta t}=k\lambda_{p}-\frac{hA\delta }{2Kl}\alpha$$

When the driving arm reaches thermal equilibrium, $\frac{\Delta T}{\Delta t}=\;0$

(9) $$\alpha_{e}=\frac{2kKl}{hA\delta }\lambda_{p}$$

where *α*_e_ is the bending angle of the bimetal sheet at thermal equilibrium. Therefore, when *λ*_p_ changes very little per step in the circular path, the time interval (10 s) is sufficient for the bending actuator to reach a new thermal equilibrium state. Therefore, the bending angle of the driving arm *α* is proportional to the projected length of the driving arm in the E‐plane.

To sum up, the control process of the parallel robot is as follows: change the direction of polarization *θ*_p_ of the microwave, so the *λ*_p_ of the driving arm changes, resulting in the change of received power of the microwaves. When it gradually reached a new thermal equilibrium state, the driving arm bent to a new angle *α*_e_, as shown in process (10)

(10) $$\theta_{p}\to \lambda_{p}\to T\to \alpha_{e}$$

The inverse solution in the triangular path is shown in Figure 4E (top), where the bending angle of each driving arm sequentially reaches its maximum value. In the experiment, the variation in *λ*_p_ was a discrete function (Figure 4E, bottom) owing to the large step size (rotates 60° per step) when *θ*_p_ was rotated from 0° to 180°. The time of each interval is shown in Figure 4C (right).

By comparing the inverse solution *α* with the change of the *λ*_p_, we see that *α* < *α*_e_ in the whole step due to the large change of *λ*_p_, which causes the change of *α* to be delayed by one step relative to that of *λ*_p_. Noteworthily, the process of *θ*_p_ varied from 0° to 60° in Figure 4E (bottom) representing the endpoint of the parallel robot moving from the center of the triangular path to the starting point (blue point) in the desired path (Figure 4E, right), which process the inverse solution did not have. Therefore, *θ*_p_ varied from 60° to 180° in the inverse solution.

The control process in the triangular path was as follows: when *θ*_p_ = 0°, we get *λ*_p_ = (*λ*_p1_,*λ*_p2_,*λ*_p3_) = (1, 0.5, 0.5), and (*α*_e1_ − *α*_1_) > (*α*_e2_ − *α*_2_) = (*α*_*e*3_ − *α*_3_). Therefore, the temperature of Arm 1 would increase significantly compared with temperature of Arms 2 and 3. After 20 s, the bending angle of Arm 1 (*α*_1_) was larger than that of the other two arms, resulting in the endpoint moving up to the blue point (Figure 4E, right). When *θ*_p_ rotated to 60°, *λ*_p_ = (0.5, 1, 0.5), *α*_1_ > *α*_e1_, *α*_2_ < *α*_e2_. Therefore, *α*_1_ began decreasing and *α*_2_ began increasing. After 30 s, *θ*_p_ was rotated to 120°, *λ*_p_ = (0.5, 0.5, 1), *α*_2_ > *α*_e2_, and *α*_3_ < *α*_e3_. Thus, *α*_2_ began decreasing, *α*_3_ began increasing, and *α*_1_ was basically unchanged. The result shows that a single microwave can effectively control the parallel robot through the polarization direction, which addresses one of the main challenges of microwave actuation.

### 2.3 Ability of Microwave‐Diving in a Closed Room

Except for metals, microwaves have high penetration into ceramics, plastics, glass, and polymers, etc. When the operating target is in the container of these materials, the microwave emitting device can be arranged flexibly outside the container (Figure 4F), whereas light, ultrasonic, or magnetic are hard to realize. To demonstrate this characteristic, we controlled the parallel robot to perform a circular path with *P* = 600 W, *D* = 450 mm in an enclosed space‐nontransparent acrylic box (Figure 4G and Movie S3, Supporting Information). The camera side and rear side of the acrylic box were transparent, so we could observe the motion and path of the parallel robot; meanwhile, the driver side was nontransparent. It is difficult for other wireless driving techniques to take into account both the far distance and confined spaces. Compared with free space, the path of the parallel robot in the acrylic box was not remarkably damped. This unique ability of microwave‐driving provides a potential solution for the controllable reactant release in a closed reactor.

### 2.4 Miniaturization of the MWD Robot

**Figure 5:**
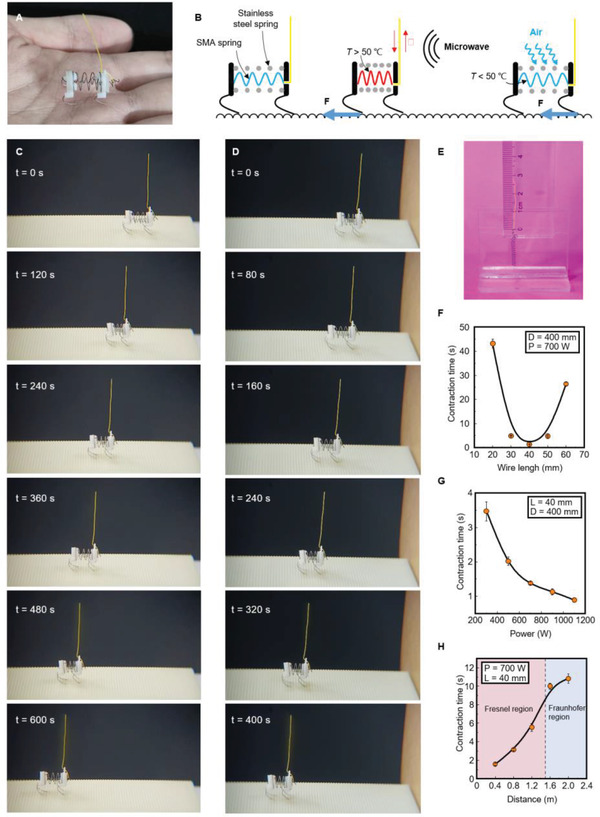
Quadruped crawling robot based on a retractable actuator. A) Photo of insect‐scale quadruped crawling robot based on retractable actuator. The robot is 15 mm long and weighs only 0.42 g. B) Schematic showing the principle of quadruped robot. C) Photographic sequence of the quadruped robot crawling on an undulation surface powered by ambient microwaves with *P* = 700 W, *D* = 400 mm. D) Sequence of image showing the crawling process of the quadruped robot behind an obstacle (cardboard box characterized by 130 mm × 130 mm × 65 mm) with *P* = 700 W, *D* = 400 mm. E) Image showing the retractable actuator composed of a wire and an SMA spring in series. F) Contraction time of the SMA spring versus the wire length under microwaves with *P* = 700 W, *D* = 400 mm. G) Relationship between contraction time of the SMA spring and the transmitting power *P* of the microwave. H) Contraction time of the SMA spring as a function of distance *D* between retractable actuator and the wave port. Error bars in (F to H) represent the SD of the averaged values from six measurements. There is an interval of 30 s for air cooling between two adjacent groups of experiments.

The design of the robot of miniaturization and lightweight can meet many engineering needs, such as small pipe inspection (household plastic water pipes), debris reconnaissance, and medical robots, which are the main challenges in the robotics field now. Compared with battery‐powered robot, MWD robot has no electronic systems, which made it easier to be lightweight and miniaturized. To demonstrate the advantage of MWD robots in miniaturization, we propose an insect‐scale quadruped crawling robot with a retractable actuator. The robot is ≈15 mm in length and 0.42 g in weight (**Figure** **5**A and Figure S5, Supporting Information). The retractable actuator was composed of a wire and an SMA spring in series. The wire could act as an antenna and would convert the microwave energy into electrical energy for the SMA spring when it was parallel to the E‐plane.

Figure 5B shows the schematic of the motion principle of the quadruped robot. The crawling process of the prototype for one period was as follows: first, due to the small elasticity modulus of the SMA spring at room temperature, an SMA spring and a stainless‐steel spring constituted an antagonistic structure, making the robot at an elongated state. Second, when the microwave irradiated the quadruped robot, the wire would offer electric power to the SMA spring, causing it to generate Joule heat. When the SMA spring reached its phase‐transition temperature (50–60 °C), the quadruped robot would shrink against the stainless‐steel spring. The legs made of metal wires on the quadruped robot bent backward, so the front leg of the robot would hook on the wave surface and generate a force F when the robot contracted, resulting in a forward movement of the robot. Finally, when the antenna stopped radiating or the polarization direction of microwaves was perpendicular to the wire, the electric energy on the SMA spring would disappear and the SMA spring would gradually cool down to room temperature. As a result, the elastic modulus of the SMA spring would be less than that of the stainless‐steel spring, and the robot would stretch under the restoring force of the stainless‐steel spring. In this process, back legs of the robot generated a force F, and front foots moved forward. Repeating the above process, the robot could achieve continuous crawling with *D* = 400 mm and *P* = 700 W (Figure 5C and Movie S4, Supporting Information). Even though the propagation route of microwaves was obscured by a cardboard box characterized by 130 mm×130 mm×65 mm, the robot maintained the same crawl movement (Figure 5D and Movie S5, Supporting Information), which demonstrated the good adaptability of the MWD robot in confined spaces.

We know that the received power of the monopole antenna is related to several factors, including the gain of the monopole antenna, the power of the microwave, and the distance between the transmitting antenna and the receiving antenna. Among them, the gain of the monopole antenna is related to the length‐to‐wavelength ratio of the antenna. Therefore, we studied the characteristics of the retractable actuators about the wire length *L*, distance *D*, and transmitting power *P*.

We bonded the actuator to a plastic ruler using transparent tape (Figure 5E). Because of the exceedingly fine diameter of the SMA wire and the impact of the electromagnetic interference, it was not possible to place electronic instruments such as infrared cameras close to the SMA spring. So it would be difficult to accurately measure the temperature of the SMA spring. Instead, we used a camera to record the contraction time of the SMA spring to represent the heating rate.

Figure 5F shows the contraction time of a 15 mm SMA spring versus the wire length *L* under ambient microwaves with *D* = 400 mm and *P* = 700 W. The experiment result shows that the received power of the actuator reached its maximum when the actuator's total length was equaled to 55 mm (*L* was ≈40 mm), which was smaller than the theoretical value (76.25 mm) due to the influence of the inductance of the SMA spring.

Figure 5G shows the relationship between the transmitting power *P* and contraction time with *D* = 400 mm and *L* = 40 mm. It can be seen from the figure that the actuator could react within 4 s when the power was less than 400 W. Moreover, the retractable actuator had a wide effective working range (Figure 5H). When the actuator was 2 m away from the antenna, in the Fraunhofer region, the SMA spring could still complete contraction within 11 s. The result shows the advantages of microwave‐driven robots in miniaturization and light‐weighting compared battery‐driven robots, demonstrating their potential in medical robots and small detection robots.

### 2.5 Group‐Driving Ability of Microwave

**Figure 6:**
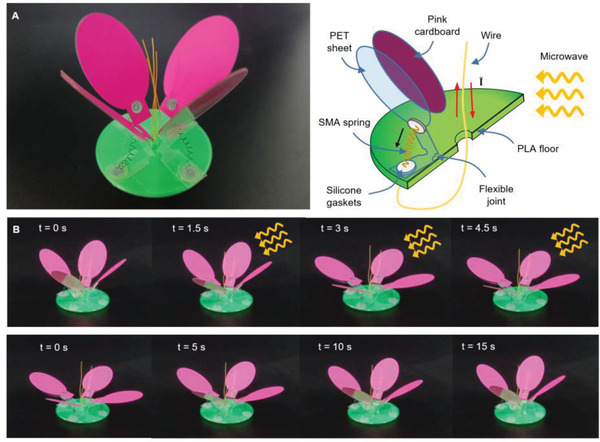
Flower‐like robot based on four retractable actuators. A) Structure of the flower‐like robot. B) Flowering and recovery process of the flower‐like robot.

To demonstrate the multiobjective driving ability of microwaves, we designed a flower‐like robot, containing four retractable actuators, which can simulate the nastic movement of the flowers (**Figure** **6**A). The structure and motion principles of the flower‐like robot are shown in Figure 6A (right). The four “petals” were fabricated from PET sheet and pink cardboard. The SMA spring was attached to silicone gaskets which adhered to the PET sheet. The design could prevent PET sheets from being burned by SMA springs. The wire was attached to the bottom side of the SMA spring and stretched up through the hole at the center of the polylactic acid (PLA) floor like a “stamen.” When microwaves radiated on the flower and the polarization direction was parallel to the stamens, the wire converted microwave energy into electrical energy (red arrow), heating the SMA spring and folding (black arrow) the PET sheet via the flexible joint. When microwaves stopped irradiating, the SMA spring gradually cooled down and removed the pulling force on the petals, resulting in all the “petals” closing under the restoring force of PET sheets. Figure 6B shows the photo sequences for the blooming (top) and closing (bottom) processes of the flower‐like robot, respectively (see Movie S6, Supporting Information). When microwaves radiated on the flower, the four “petals” bloomed almost simultaneously. In conclusion, like magnetic actuation, microwave actuation also has the capability of swarm‐driving.

## 3 Conclusion

In this paper, we presented a microwave‐driven robot controlled by tuning the polarization direction of microwaves. At the same time, we demonstrated and supported the idea of path control experimentally and theoretically. Moreover, we studied the heating mechanism of the wave‐absorbing sheet under different microwave components and optimized the geometry of the bending actuator to have a sufficient response rate. The heating effect of the electric field on the bending actuator is more obvious than that of the magnetic field at the optimal length of the wave‐absorbing sheet. Although the circular and triangular paths of the parallel robot can be realized, there is an error in the actual motion of the endpoint compared with the ideal path. Through analysis, we think the main path error of the parallel robot comes from the manufacturing error of each bending actuator and the assembly error among the PET arm, the bending actuator, and the frame, which results in inconsistent motion of each driving arm. By controlling the power and irradiation time of the microwave, the robot can work within a safe temperature range (−20–120 °C) to achieve its long‐term stability.

Furthermore, we additionally demonstrated the potential of microwave‐driven robots in miniaturization, lightweight, and swarm‐driving. The insect‐scale crawling robot, with a weight of only 0.42 g, is the smallest robot powered by microwaves. The feature gives microwave‐driven robots an application prospect, such as targeted drug delivery in cancer treatment or tasks in closed containers or lathy pipelines. However, the real‐time control of the microwave power and frequency has not been exerted over the robot. The control will further improve the precision and the functionality of the robot. For the microwave‐driven robots, how to cool the actuators is the key problem. We can optimize the cooling rate by optimizing the size of the actuator (e.g., using a thinner SMA spring or bending actuator) or adding a heat transfer system. In conclusion, our results show that microwaves have far‐field driving capability, swarm‐driving capability, and adaptability to confined spaces. Furthermore, the microwave as a wireless actuation method can not only provide energy but also offer controllability.

Looking forward, the penetrating ability of microwaves can effectively drive targets in nonmetallic containers. In metal pipes or containers, we can use waveguides to steer electromagnetic waves, achieving long‐range propagation of microwaves to drive targets. These advanced ideas can expand the range of design, motion modalities, and functions of microwave‐driven robotic systems, facilitating practical applications in healthcare, operation in microwave‐exposed environments, and detection of enclosed environments and others.

## 4 Experimental Section

### Bending Actuators and Parallel Robots

The magnetron of the horn antenna was an air‐cooled Samsung magnetron OM75P with a frequency of 2465 MHz. The wave‐absorbing material used in this study was an ultra‐thin magnetic alloy composite material with excellent absorption capacity for high‐frequency electromagnetic waves. The thickness of the wave‐absorbing material was 0.3 mm, the permeability was 120 (at 3 MHz), the range of available temperature was −25 to 120 °C, and the application frequency was 10 MHz to 6 GHz. The bimetallic strip's model number was 5J20110, and its linear temperature range was −20 to 150 °C. The working temperature of the robot should not be exceeded the allowable temperature range of the wave‐absorbing material and bimetal, otherwise it would damage the microwave actuator and affect its long‐term stability.

The fabrication process of the parallel robot was as follows: the wave‐absorbing material was cut into a wave‐absorbing sheet (40 mm × 10 mm) using a laser cutter. Then, the trapezoidal bimetallic sheet (3 mm × 7 mm × 40 mm) was stuck onto the wave‐absorbing sheet with its active deformation layer in contact with the wave‐absorbing material to form a driving arm. Finally, one end of the driving arm was fixed on the acrylic frame using screws and an acrylic sheet, and the other end was glued to the PET arm using silicone glue 988. The acrylic frame was cut from a 3.5 mm thick acrylic sheet, and the PET arm was cut from a 0.4 mm thick PET sheet using a laser cutter.

### Retractable Actuators and Quadruped Robots

The retractable actuator in the quadruped crawling robot was composed of a conductive wire and a commercial SMA spring, which could shrink after heating and bounce back to its original length after cooling. The two‐way SMA spring was fabricated from Ni‐Ti alloy with a wire diameter of 0.2 mm and a spring diameter of 2.2 mm. The phase transition temperature of the SMA spring was 50–60 °C. The signal wire acting as an antenna had a diameter of 0.3 mm and consisted of 19 strands of 0.04 mm silver‐plated copper wires.

The quadruped robot (Figure S5A, Supporting Information) was composed of an SMA spring (Figure S5B, Supporting Information), two structural parts (Figure S5B,F, Supporting Information), a stainless steel spring (Figure S5D, Supporting Information), a wire (Figure S5E, Supporting Information), two silicone gaskets (Figure S5G, Supporting Information), and four legs composed of iron wires (Figure S5H, Supporting Information). These components were grouped into three categories based on their functionality.

1) The trunk of the robot, including a stainless‐steel spring, two silicone gaskets, and two structural parts. The stainless‐steel spring was fixed to the structural parts by interference fitting; and then, glue was used to reinforce the junction between the stainless‐steel spring and the structural parts. The stainless‐steel spring had five turns, and had a radius of 7 mm, a length of 10 mm, and a wire diameter of 0.3 mm. The silicone gasket was cut from a 1.5 mm thick silicon rubber sheet and bonded to the structural parts with glue.
2) The driving module, including a 12 mm two‐way SMA spring and a 40 mm silver‐plated wire. The SMA spring was installed on the silicone gaskets and was coaxial with the stainless‐steel spring. Silicone gaskets prevented structural parts from being burned by the SMA spring. The wire, passed through the rear structural part to receive the microwave, was wound around the tail of the SMA spring.
3) Legs, including wires whose diameter was 0.2 mm, were fabricated from iron. The iron wires were installed on the front and rear structural parts and were bent backward.

## Conflict of Interest

The authors declare no conflict of interest.

## Author Contributions

J.Z. and Z.X. conceived the idea. Z.X. accomplished some preliminary works. Y.L. carried out the experiments and wrote the first manuscript. J.Z. and Z.X. directed this project and revised the manuscript. L.S., P.Y., and J.W. directed the antenna theory.

## Supporting information

Supporting Information

Supplemental Movie 1

Supplemental Movie 2

Supplemental Movie 3

Supplemental Movie 4

Supplemental Movie 5

Supplemental Movie 6

## Data Availability

The data that support the findings of this study are available in the supplementary material of this article.
