# Supplementary material for: Multi‐Degree‐of‐Freedom Robots Powered and Controlled by Microwaves
Source: Adv Sci (Weinh). 2022 Aug 19;9(29):2203305. doi: 10.1002/advs.202203305 (PMC9561789; doi:10.1002/advs.202203305)
Supplement: Supplementary file 1 — Supporting Information [file ADVS-9-2203305-s002.pdf]

ADVANCED SCIENCE
Open Access

Supporting Information

for *Adv. Sci.*, DOI 10.1002/advs.202203305

Multi-Degree-of-Freedom Robots Powered and Controlled by Microwaves

Yongze Li, Jianyu Wu, Peizhuo Yang, Lizhong Song, Jun Wang, Zhiguang Xing and Jianwen Zhao**

Supplementary Materials for

Multi-degree-of-freedom Robots Powered and Controlled by Microwaves

Authors:

Yongze Li[1], Jianyu Wu[1], Peizhuo Yang[2], Lizhong Song[2], Jun Wang[2], Zhiguang Xing[1]*, Jianwen Zhao[1]*

Affiliations:

[1]Department of Mechanical Engineering, Harbin Institute of Technology; Weihai 264209, China.

[2]School of Information Science and Engineering, Harbin Institute of Technology Weihai; Weihai 264209, China.

*Corresponding author. Email: zhiguangx@hit.edu.cn (Z. Xing); zhaojianwen@hit.edu.cn (J. Zhao).

This PDF file includes:

Supplementary Text
Text S1 to S2
Figs. S1 to S5
Tables S1 to S2
Movies S1 to S6

Other Supplementary Materials for this manuscript include the following:

Movies S1 to S6

Supplementary Text

Text S1. Calculation of the field range of the horn antenna

The outer field space of the antenna can be divided into three regions: Fraunhofer, Fresnel, and induced near-field regions. In the induction near-field region, the induction field is larger than the radiation field, and the virtual power of the round-trip oscillation is larger than the actual power propagated along the radial direction. In the radiative field regions (Fraunhofer and Fresnel regions), the radiative energy dominates. Therefore, the robot should be placed in the radiative field region. The range of the three types of spaces is given by

$$R_1 = 0.62\sqrt{\frac{S^3}{\lambda}} \tag{1}$$

$$R_2 = \frac{2S^2}{\lambda} \tag{2}$$

where R_1 is the radius of the induced near-field region, R_2 is the radius of the Fresnel region, S is the maximum value of wave port size, and λ is the wavelength. In this paper, S = 300 mm, $\lambda = 120$ mm. Therefore, the Fresnel region is within the range of 291 mm to 1470 mm, and the Fraunhofer region is beyond 1470 mm.

Text S2. Inverse solution of the parallel robot

Assuming that the driving arm 1 rotates in the XOZ plane, and the coordinates of the connecting point $C_i(i = 1, 2, 3)$ between the driven arm and moving platform are (x_i', y_i', z_i'). Thus, the driving arms rotate around the Z-axis to the XOZ plane:

$$C_1' = C_1 = (x_1, y_1, z_1)$$
$$C_2' = \mathrm{Rot}(z, 240°)C_2 = (x_2, y_2, z_2)$$
$$C_3' = \mathrm{Rot}(z, 120°)C_3 = (x_3, y_3, z_3)$$

The inverse solution for the Delta parallel robot is expressed as follows:

$$\theta_i = -\,\mathrm{asin}\left(\frac{(x_i+r-R)^2+y_i^2+z_i^2+L^2-l^2}{2L_1\sqrt{z_i^2+(x_i+r-R)^2}}\right) + \varphi_i \quad (i = 1,2,3) \tag{3}$$

$$\varphi_i = \mathrm{asin}\left(\frac{R-x_i-r}{\sqrt{z_i^2+(x_i+r-R)^2}}\right) \quad (i = 1,2,3) \tag{4}$$

where θ_i is the angle of the driving arms, L = 30 mm is the length of the driving arm, l = 90 mm is the length of the PET arm, r = 1.5 mm is the radius of the moving platform, and R = 50 mm is the radius of the static platform.

The inverse solution of the kinematics of the Delta parallel robot is shown in Figure 1 (G and I). The inverse solution trajectory is on the right-hand side of the graph, and the blue point is the track start point. It should be noted that the inverse solution trajectory is only an approximation of the actual motion trajectory of the Delta parallel robot. In practice, the endpoint begins from the center point to the intended trajectory instead of the blue point in the inverse solution trajectory. However, it is sufficient for the qualitative analysis.

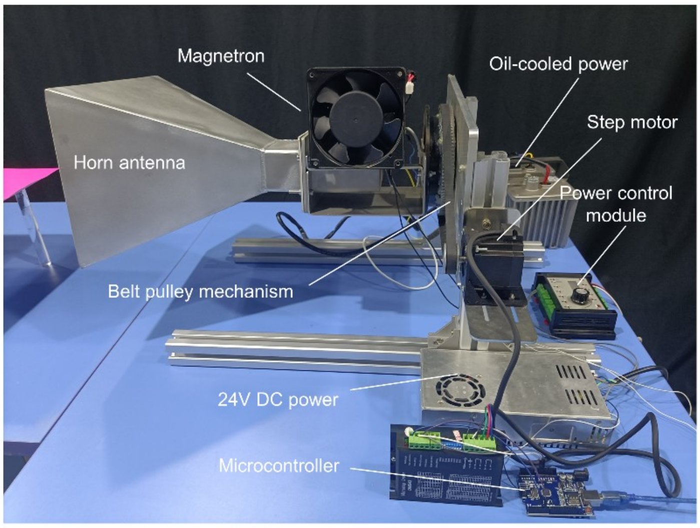

Figure S1.

Microwave transmitter. The microwave transmitting device was composed of a magnetron, a horn antenna, a oil-cooled power supply, a 24 VDC power supply, a power control module, a step motor, a belt pulley machanism and a microcontroller (Arduino UNO). The magnetron was an air-cooled Samsung magnetron OM75P with a frequency of 2465 MHz and power of 1050 W. The size of the waveguide conversion interface was 90 mm×50 mm, and the horn antenna was 300 mm long, 230 mm wide, and 233.2 mm high. To realize the adjustment of the direction of polarization of microwaves, the microcontroller controlled a stepper motor to drove a pulley to rotate the antenna.

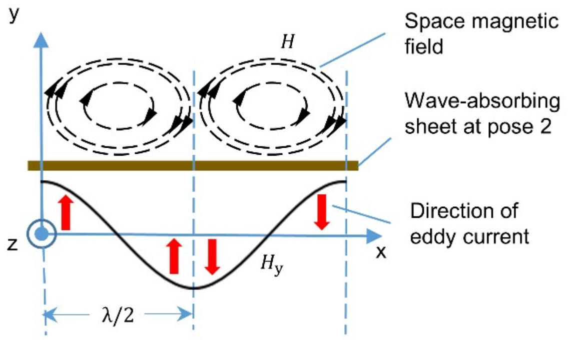

Figure S2.
Eddy current generated by the wave-absorbing sheet at pose 2. The magnetic field generated by the horn antenna in space is a vortex magnetic field, and H_y can be approximately considered a sine function. According to Lenz's law, the eddy current length on the wave-absorbing sheet of pose 2 is half-wavelength.

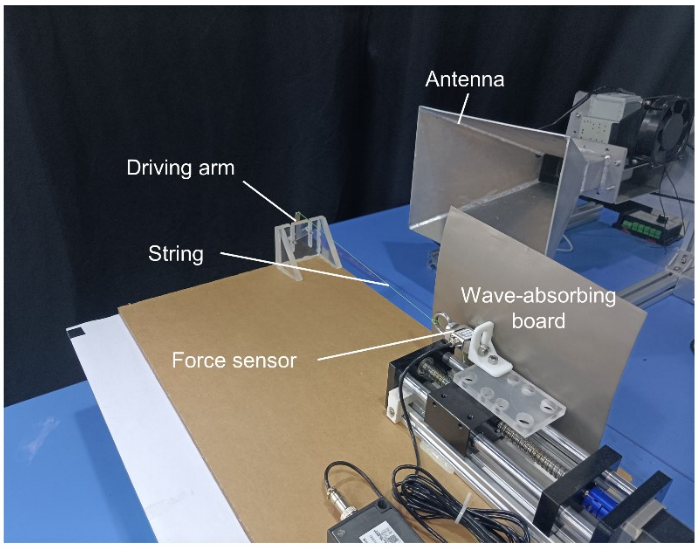

Figure S3.

Photo of tensile force measuring device. The driving arm was fixed on the acrylic plate and connected to a tension sensor outside the radiation field by a string. The tension sensor was a 0–500 g miniature tension sensor (JLBS-M2). The tension sensor was fixed on the leadscrew and could move back and forth. The screw system and the sensor were shielded with an absorbing material to prevent microwaves from interfering with the measurement system. We used Arduino UNO to collect and process data.

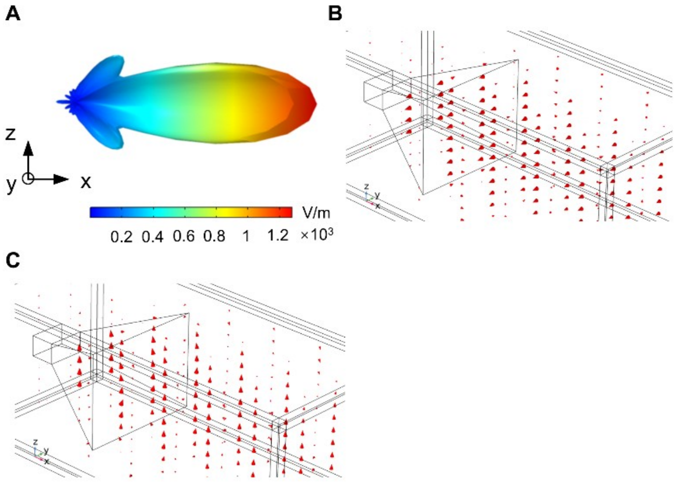

Figure S4.

COMSOL simulation for horn antenna. A) Radiation far-field pattern of the horn antenna. The directivity was 15.86 dB. B) Illustration of the magnetic field vector in the radiated field. C) Illustration of the electric field vector in the radiated field.

The waveguide of the horn antenna was 9 cm long and 5 cm wide. The model was established in the multiphysics simulation software COMSOL according to the parameters in Table S2. Figure S4A shows that the main lobe of the microwave along the normal direction (x-axis) of the horn antenna was significantly larger than the side lobes on both sides. The directivity coefficient calculated by COMSOL was 15.86 dB. Figure S4 (B and C) show the magnetic and electric field vectors in the radiation field, respectively.

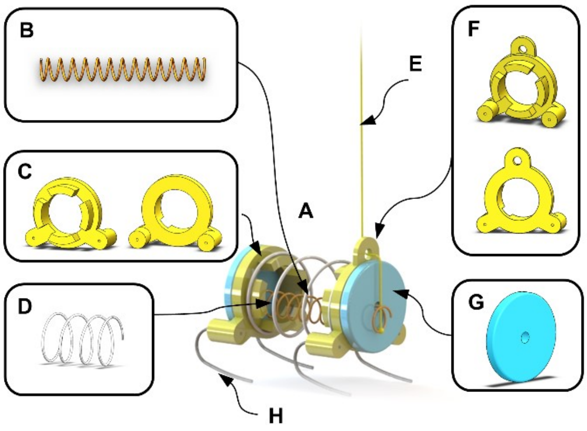

Figure S5.

Structure of the quadruped crawling robot. A) Schematic of microwave-driven quadruped robot. B) SMA spring. C) Front structure part. D) Stainless steel spring. E) Wire. F) Rear structure part. G) Silicone gasket. H) Iron wire.

Table S1.

Composition of flexible electromagnetic wave absorbing film .

Product Composition	Proportion	CAS Number
Polyurethane	5%-30%``	51852-81-4
Ferrimag	50%-90%	7439-89-6
Acrylic polymers	0.01%-1%	35239-19-1
PET	0.05%-1%	25038-59-9

Table S2.

COMSOL parameter configuration.

Type of port	Mode type	Mode number	Port input power (W)	Frequency (GHz)
Rectangle	TE	10	700	2.45

Movie S1.

Circular path control of parallel robots.

Movie S2.

Triangular path control of parallel robots.

Movie S3.

Robotic motion in confined spaces.

Movie S4.

Crawling of the microwave-driven quadruped robot.

Movie S5.

Quadruped robot crawling in an obstacle course.

Movie S6.

Flower-like robot.
